# Efficient Isolation of Lymphocytes and Myogenic Cells from the Tissue of Muscle Regeneration

**DOI:** 10.3390/cells11111754

**Published:** 2022-05-26

**Authors:** Yasuo Kitajima, Ryoka Tsukahara, Shohei Nakamoto, Tomoharu Yasuda

**Affiliations:** Department of Immunology, Graduate School of Biomedical and Health Sciences, Hiroshima University, 1-2-3 Kasumi, Minami-ku, Hiroshima 734-8551, Japan; b182693@hiroshima-u.ac.jp (R.T.); b180294@hiroshima-u.ac.jp (S.N.); yasudat@hiroshima-u.ac.jp (T.Y.)

**Keywords:** muscle regeneration, lymphocytes, myogenic cells, Percoll, CD45 beads

## Abstract

Isolation of both lymphocytes and myogenic cells from muscle tissue is required for elucidating the cellular and molecular mechanisms of muscle regeneration. Here, we aimed to establish an optimal method obtaining a high yield of lymphocytes during muscle regeneration. After the muscle injury, we observed higher infiltration of lymphocytic cells in the muscle on day 3 after injury. Then, we compared two different white blood cell isolation methods, the Percoll gradient and CD45-magnetic bead methods, to assess the percentage and number of T and B cells. Flow cytometry analysis showed that the CD45-magnetic bead method has a better efficiency in isolating CD4^+^, CD8^+^ T cells, and B cells from injured muscle tissues of wild-type and *mdx* mice than that by the Percoll gradient method. Moreover, we found that the CD45-negative fraction from wild-type and *mdx* mice includes myogenic cells. In conclusion, we report that the CD45-magnetic bead method is suitable to isolate T and B cells during muscle regeneration with higher purity and yield and can also isolate myogenic cells within the same sample. This method provides a technical basis for further studies on muscle regeneration, involving lymphocytes and muscle cells, with a wide range of clinical applications.

## 1. Introduction

Skeletal muscle is the largest tissue in the body, accounting for approximately 40% of body weight [1], and has an extremely high regenerative capacity attributed to the presence of muscle stem cells, also known as muscle satellite cells, present between the basal lamina and plasmalemma of myofibers [2]. In the intact state, satellite cells remain quiescent, but they are activated in response to muscle injury, leading to their proliferation and differentiation into mature muscle fibers [3,4,5,6]. Muscle stem cells are essential for skeletal muscle homeostasis and muscle regeneration throughout life [3,7,8,9]. Therefore, understanding the dynamics of muscle satellite cells will provide valuable insights into the mechanisms of muscle regeneration during disease and aging.

In addition to satellite cells, other cell types are involved in muscle regeneration [10]. Lymphocytes immediately infiltrate the muscle tissue in response to muscle injury [11,12]. CD4 and CD8 are the membrane proteins [13,14,15] of helper and cytotoxic T cells, respectively, while B220 and CD19 [16,17] are cell surface markers of B cells. The CD4^+^ helper T cells help B cells to generate high-affinity antibodies [18]. The CD8^+^ cytotoxic T cells play an important role in immune defense against intracellular pathogens, such as viruses and bacteria, as well as tumors [19]. Single-cell analysis has reported that CD4^+^ T cells, CD8^+^ T cells, and B cells are increased during muscle regeneration [10,20,21], suggesting their involvement in the process. In CD8-deficient mice lacking MHC class I-restricted cytotoxic T lymphocytes, the regenerating muscle fibers are smaller, and fibrosis increases after muscle injury [22]. In Rag2 and *γ*-chain double knockout mice (lacking T cells, B cells, and natural killer lymphocytes), the regenerating muscle fibers are smaller owing to the impairments in satellite cell expansion [23]. In addition, regulatory T cells are critical regulators of immune responses [24], and their ablation impairs muscle regeneration after muscle injury [25]. These findings indicate, in part, that skeletal muscle regeneration occurs through interactions between lymphocytes and muscle cells; however, data supporting this hypothesis is lacking. To elucidate the details of this interaction in muscle regeneration, it is important to establish an efficient method for collecting a small number of lymphocytes, such as T and B cells.

However, to date, an optimal method for the fractionation of lymphocytes in skeletal muscle has not been established. Many studies, including ours, have employed enzymatic treatment using collagenase to process skeletal muscle to obtain mononuclear cells [26,27,28,29,30,31,32,33]. Isolates obtained post-collagenase treatment of skeletal muscle contain a large amount of myofibrillar debris [34]. These debris make the isolation of cells from muscle less efficient and were therefore removed using Percoll density gradient centrifugation [35,36] and fluorescence-activated cell sorting [37]. The Percoll gradient method is widely used for isolating lymphocytes from tissues other than the muscles [38,39,40]. The CD45-magnetic bead isolation method is another alternative for lymphocyte isolation [41,42,43]. Isolation using CD45 beads would lead to the removal of debris from the muscle generated by enzymatic treatment, and the subsequent use of a cell sorter could aid in concentrating the lymphocytes. Establishing an efficient method for isolating lymphocytes from muscles using these techniques would assist in the detailed study of the involvement of lymphocytes and myogenic cells in muscle regeneration.

In this study, we aimed to compare the Percoll gradient and CD45-magnetic bead isolation methods for the isolation of lymphocytes during skeletal muscle regeneration to obtain a pure and high yield of lymphocytes.

## 2. Materials and Methods

### 2.1. Mouse Strains

All the animal experiments were approved by the Experimental Animal Care and Use Committee of Hiroshima University (Ref. No. A21-103). C57BL/6J and *mdx* mice were acquired from CLEA Japan (Tokyo, Japan) and maintained in a controlled environment with free access to food and water. All the mice used in these experiments were males aged 2–3 months.

### 2.2. Muscle Injury

A Cardiotoxin (CTX, Sigma-Aldrich, St. Louis, MO, USA, Cat. No. L8102)-induced muscle injury model was created as described previously [44]. CTX was dissolved in 0.9% NaCl to a final concentration of 10 μM and stored at −80 °C until use. Next, 50 μL of CTX (10 μM) was injected percutaneously into the tibialis anterior and gastrocnemius muscles of anesthetized mice. The needle for injection was inserted into the center of these muscles to avoid going too deep into the muscle. Mice were allowed to recover for 3 and 14 days after injury. The injected and non-injected muscles were harvested for flow cytometry analysis.

### 2.3. Antibodies

The following antibodies were used from BioLegend (San Diego, CA, USA): PE-conjugated anti-Sca-1 (Cat. No. 122507, 1:400 dilution), anti-CD31 (Cat. No. 102407, 1:400 dilution), and anti-CD45 antibodies (Cat. No. 103105, 1:400 dilution); biotin-conjugated anti-Vcam1 antibody (Cat. No. 105703, 1:200 dilution); BV650-conjugated streptavidin (Cat. No. 405231, 1:200 dilution); BV605-conjugated anti-CD19 antibody (Cat. No. 115539, 1:400 dilution); and PE/Cy7-conjugated anti-CD4 antibody (Cat. No. 100421, 1:400 dilution). PE-conjugated anti-B220 antibody (Cat. No. 553089, 1:400 dilution), APC-conjugated anti-CD8 antibody (Cat. No. 553035, 1:400 dilution), and anti-CD16/CD32 antibody (Cat. No. 553142, 1:100 dilution) were obtained from BD Biosciences (Franklin Lakes, NJ, USA).

### 2.4. Isolation of Mononuclear Cells in Muscle 

The tibialis anterior and gastrocnemius muscles of the mice were dissected and collected in phosphate-buffered saline (PBS). The muscles were placed in a new Petri dish containing PBS. Any visible fat and nerve deposits were removed using forceps. The muscles were placed on a plastic plate and minced into a paste using small surgical scissors until no visible muscle deposits remained. The paste was then transferred to a tube containing 0.2% type II collagenase (Worthington Biochemical Corp. Lakewood, NJ, USA, Cat. No. CLS2) dissolved in Dulbecco’s modified Eagle’s medium (DMEM). Approximately 3 mL of collagenase solution for 1 g muscle tissue was prepared. The paste was incubated at 37 °C for 60 min on a shaker. The minced muscle tissue was passed through an 18G syringe needle several times and then incubated at 37 °C for 30 min. The homogenate was collected using an 18G syringe needle, dissolved in 40 mL of PBS, then filtered using a 40 μm nylon mesh strainer. The flow-through was collected in a 50 mL conical tube and centrifuged at 500× *g* for 5 min at 4 °C. The supernatant was discarded, and the mononuclear cells were resuspended in PBS.

### 2.5. Separation of Mononuclear Cells Using Percoll and CD45 Beads

The enzyme-treated mononuclear cells were divided equally into three groups: control, Percoll, and CD45 bead groups. For the control group, the mononuclear cells were stained directly, whereas the Percoll gradient and CD45-magnetic bead methods were used for the Percoll and CD45 bead groups, respectively. 

The Percoll gradient method was performed to isolate the leukocyte fraction. First, mononuclear cells were resuspended in 5 mL of 40% Percoll (Cytiva, Marlborough, MA, USA, Cat. No. 17544501), layered on 5 mL of 80% Percoll, and centrifuged at 1400× *g* for 15 min at 4 °C. The lymphocytes in the layer between the 80% and 40% Percoll solutions were collected and added to 5 mL of DMEM. The solution was centrifuged at 500× *g* for 5 min at 4 °C. The supernatant was discarded, and the cells were stained.

The CD45-magnetic bead method was completed using CD45 MicroBeads (1:10; Miltenyi Biotec, Bergisch Gladback, Germany, Cat. No. 130-052-301). The CD45 beads were incubated with mononuclear cells for 15 min at 4 °C. After incubation, 5 mL of PBS containing 2% fetal bovine serum (2% FBS/PBS) was added and centrifuged at 500× *g* for 5 min at 4 °C. The supernatant was discarded, and the cells containing CD45 beads were resuspended in 2 mL of 2% FBS/PBS. CD45-positive cells containing lymphocytes were purified by positive selection using a MACS Separation LS Column (Miltenyi Biotec, Bergisch Glad-back, Germany, Cat. No. 130-042-401) and MidiMACS Separator (Miltenyi Biotec, Bergisch Gladback, Germany, Cat. No. 130-042-302) according to the manufacturer’s instructions. In brief, the LS Column was placed on the MidiMACS separator and 2 mL of cell suspension containing CD45 beads was poured into the LS Column. The cell suspension that flowed through the LS Column was centrifuged at 500× *g* for 5 min at 4 °C. The supernatant was discarded, and the cells were designated as CD45-negative cells. On the other hand, the magnetically labeled fractions were retained in the LS Column and were removed from the MidiMACS Separator. Following this, 5 mL of 2% FBS/PBS was poured into the LS Column and the cell suspension of CD45-positive cells was collected. The cell suspension was centrifuged at 500× *g* for 5 min at 4 °C. The supernatant was discarded, and the cells were designated as CD45-positive cells. The isolated CD45-positive cells containing lymphocytes and CD45-negative cells containing myogenic cells were subsequently stained.

### 2.6. Staining of Lymphocyte Populations

Cells were incubated with anti-CD16/CD32 for 10 min at 4 °C to reduce non-specific binding of the cells before staining. After incubation, 2% FBS/PBS was added, and the suspension was centrifuged at 500× *g* for 3 min at 4 °C. The supernatant was discarded, and the cells were incubated with a mixture of antibodies CD4, CD8, CD19, and B220 for 15 min at 4 °C. After incubation, 2% FBS/PBS was added and centrifuged at 500× *g* for 3 min at 4 °C. The supernatant was discarded, and the stained cells were analyzed by flow cytometry. Each lymphocyte cell population was defined as follows: CD4^+^ T cells (CD4^+^), CD8^+^ T cells (CD8^+^), and B cells (B220^+^CD19^+^).

### 2.7. Myogenic Cells Staining with CD45-Negative Cells

As described in previous studies [45], myogenic cell markers were stained with the antibodies Sca-1, CD31, CD45, and Vcam1. CD45-negative cells were incubated with a mixture of antibodies (Sca-1, CD31, CD45, and Vcam1) for 15 min at 4 °C. After incubation, 2% FBS/PBS was added, and the cells were centrifuged at 500× *g* for 3 min at 4 °C. The supernatant was discarded, and cells were incubated with a streptavidin antibody for 15 min at 4 °C. After incubation, 2% FBS/PBS was added and centrifuged at 500× *g* for 3 min at 4 °C. The supernatant was discarded, and the stained cells were analyzed by flow cytometry. The population of myogenic cells was defined as Vcam1^+^CD31^−^CD45^−^Sca1^−^ cells.

### 2.8. Flow Cytometry Analysis

Isolated cells were stained using respective antibodies and further analyzed using a CytoFLEX S flow cytometer (Beckman Coulter, Brea, CA, USA) and FlowJo v10 software (Tree Star Inc, San Carlos, CA, USA). Debris and dead cells were excluded using gates based on the forward scatter, side scatter, and propidium iodide staining.

### 2.9. Statistical Analysis

Statistical analyses were performed using GraphPad Prism version 8.0.0 for Windows (GraphPad Software, San Diego, CA, USA) to determine significant differences based on a two-tailed distribution using a Student’s *t*-test. For comparisons between more than two groups, a one-way analysis of variance (ANOVA) followed by Dunnett’s post hoc test was used. *p*-values are as indicated in each figure *p* < 0.05 (*), <0.01 (**), <0.001 (***), and <0.0001 (****). Data are represented as mean ± standard error of the mean (SEM).

## 3. Results

### 3.1. Infiltration of T and B Cells into Injured Muscle

**Figure 1:**
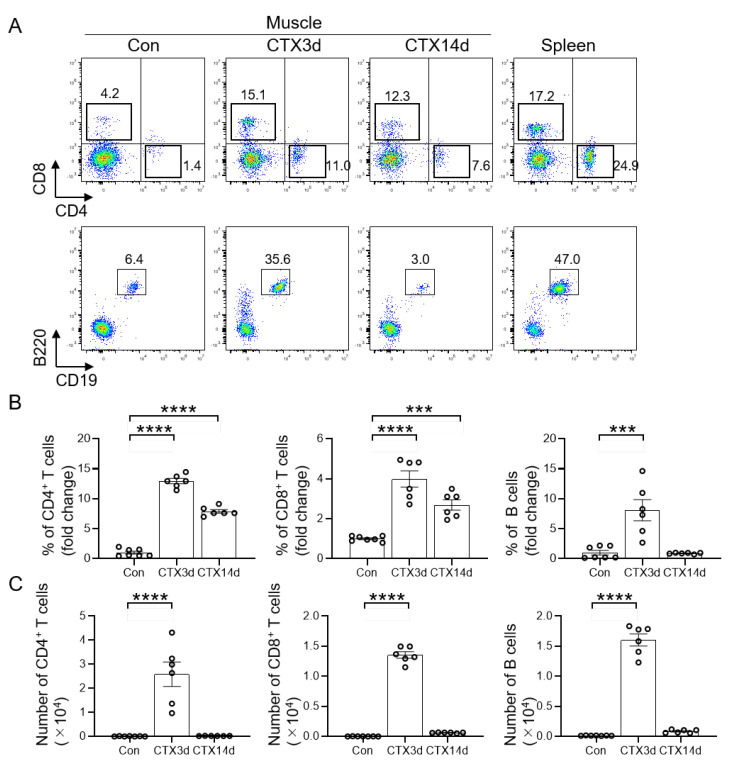
T and B cells infiltrate into injured muscle. (**A**) Flow cytometry analysis profiles of muscle-derived CD4^+^ T cells (helper T cells), CD8^+^ T cells (cytotoxic T cells), and B cells without injury, and at days 3 and 14 after injury. The gated profiles show T cells (CD4^+^ or CD8^+^) and B cells (B220^+^CD19^+^). (**B**) Fold change in the percentage of CD4^+^ T cells, CD8^+^ T cells, and B cells in the CTX3d and CTX14d relative to the control group. (**C**) The number of T and B cells in the three groups was analyzed. Data represented as the mean ± SEM (one-way ANOVA followed by Dunnett’s post hoc test: *** = *p* < 0.001, **** = *p* < 0.0001; *n* = 6–7 per group).

To understand the dynamics of lymphocytes, such as T and B cells, during skeletal muscle regeneration, CTX was injected into the muscle of the mice to induce skeletal muscle injury. The injured muscles were digested using enzymatic treatment to obtain mononuclear cells and analyzed by flow cytometry without injury and on days 3 and 14 after injury. The highest infiltration of T and B cells into the injured muscle was observed 3 days after injury, while there were very few T and B cells in the uninjured muscle (Figure 1A). Quantification of the relative percentage of CD4^+^ T cells and CD8^+^ T cells showed significantly higher infiltration on days 3 and 14 after injury compared to the uninjured muscle, whereas B cells showed a significant increase on day 3 after injury (Figure 1B). In addition, the number of T and B cells on day 3 after injury was significantly higher than that during the uninjured period (Figure 1C). These results indicated that T and B cell infiltration was the highest on day 3 of injury in muscle induced by CTX.

### 3.2. Efficient Isolation of T and B Cells from Injured Muscle Using CD45 Beads

**Figure 2:**
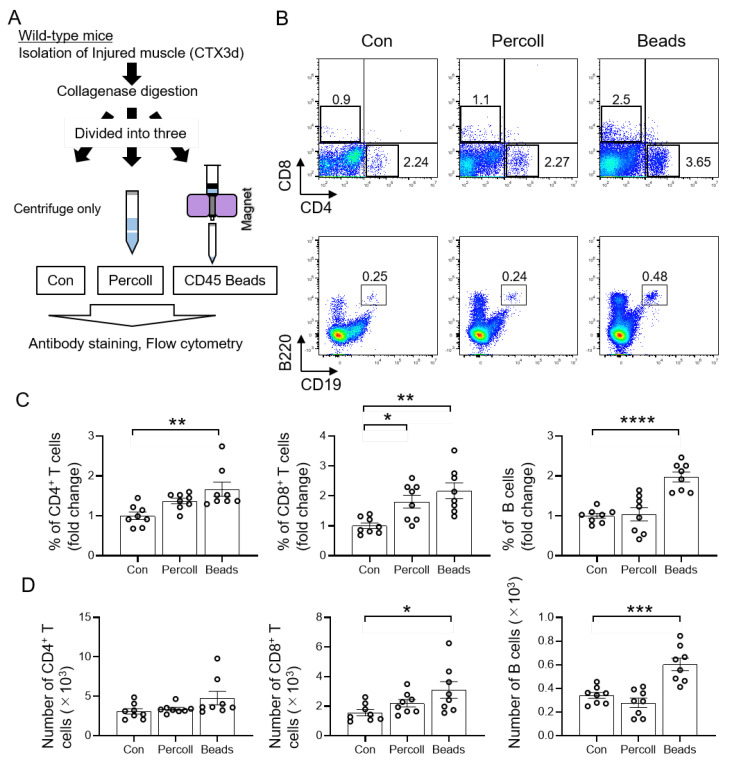
Isolation of T and B cells from injured muscle using CD45 beads is efficient. (**A**) Schematic representation of the experimental workflow for the analysis of injured muscle on day 3 after injury with Percoll and CD45 beads isolation methods in wild-type mice. (**B**) Flow cytometry analysis profiles of muscle-derived CD4^+^ T cells (helper T cells), CD8^+^ T cells (cytotoxic T cells), and B cells on day 3 after injury. The gated profiles show T cells (CD4^+^ or CD8^+^) and B cells (B220^+^CD19^+^). (**C**) Fold change in the percentage of CD4^+^ T cells, CD8^+^ T cells, and B cells in the Percoll and CD45 bead groups relative to the control group. (D) The number of T and B cells were analyzed. Data represented as the mean ± SEM (one-way ANOVA followed by Dunnett’s post hoc test: * = *p* < 0.05, ** = *p* < 0.01, *** = *p* < 0.001, **** = *p* < 0.0001; *n* = 8 per group).

To investigate the role of T and B cells in muscle regeneration, it is important to isolate these cells with high purity. Therefore, we focused on day 3 after injury, when the infiltration of T and B cells during muscle regeneration was high, and investigated efficient isolation methods using Percoll and CD45 beads (Figure 2A). Percoll is used in density gradient centrifugation to isolate lymphocytes from tissues [25,38]. In addition, CD45 beads have been widely used to isolate lymphocytes because the CD45 antigen is expressed in all cells of hematopoietic origin. Flow cytometry analysis revealed that the usage of CD45 beads as a method of isolation was effective in providing the highest percentage of CD4^+^ T cells, CD8^+^ T cells, and B cells compared to that in the control (Figure 2B,C). Further quantification of cell counts showed that the number of CD8^+^ T and B cells was significantly higher in the CD45 beads isolate than that in the control (Figure 2D). These data demonstrate that CD45 beads aid in the efficient isolation of T and B cells from injured muscles.

### 3.3. Isolation of T and B Cells by CD45 Beads in Mdx Mice

**Figure 3:**
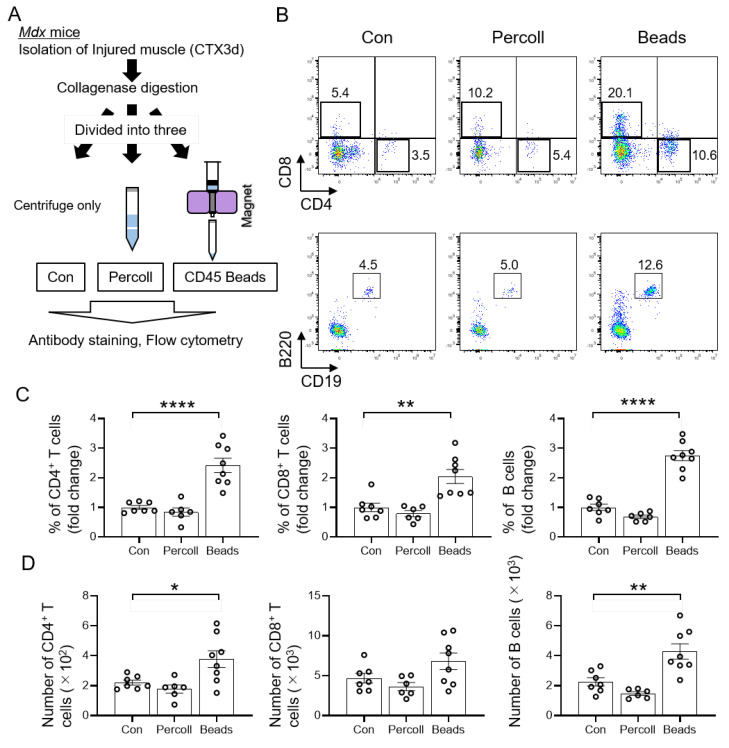
Isolation of T and B cells using CD45 beads in *mdx* mice. (**A**) Schematic representation of the experimental workflow for the analysis of injured muscle on day 3 after injury with Percoll and CD45 beads isolation in *mdx* mice. (**B**) Flow cytometry analysis profiles of muscle-derived CD4^+^ T cells (helper T cells), CD8^+^ T cells (cytotoxic T cells), and B cells on day 3 after injury. The gated profiles show T cells (CD4^+^ or CD8^+^) and B cells (B220^+^CD19^+^). (**C**) Fold change in percentage of CD4^+^ T, CD8^+^ T, and B cells for Percoll, and CD45 beads groups relative to the control group. (**D**) The number of T and B cells was analyzed. Data represent the mean ± SEM (one-way ANOVA followed by Dunnett’s post hoc test: * = *p* < 0.05, ** = *p* < 0.01, *** *= p* < 0.001, **** = *p* < 0.0001; *n* = 6–8 per group).

We further examined the methods for efficient isolation of T and B cells on day 3 after muscle injury in the *mdx* mouse model (Figure 3A). Flow cytometry analysis showed that, similar to the wild-type mice, T and B cell isolation using CD45 beads represented the cell population of *mdx* mice (Figure 3B). Quantification of the percentage of isolated T and B cells revealed that isolation using CD45 beads resulted in the highest percentage of the respective cells in the CD45 bead group compared to that in the controls (Figure 3C). In addition, CD45 beads produced the highest yield of CD4^+^ T and B cells (Figure 3D). These results indicated that the isolation of T and B cells using CD45 beads is the most efficient method for increasing both the purity and yield of lymphocytes from the muscle of wild-type and *mdx* mice.

### 3.4. Isolation of Muscle Stem Cells Using CD45 Negative Cell Population

**Figure 4:**
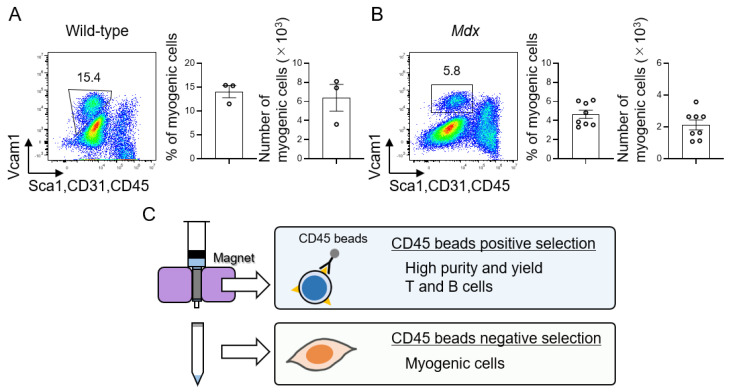
Isolation of myogenic cells using CD45-negative cells. (**A**,**B**) Flow cytometry analysis profiles of myogenic cells on day 3 after injury in (**A**) wild-type mice (*n* = 3) and (**B**) *mdx* mice (*n* = 8). The gated profiles show myogenic cells (Vcam1^+^CD31^−^CD45^−^Sca1^−^). The percentage and number of myogenic cells were analyzed. Data represent the means ± SEM. (**C**) Schematic representation of this study. Isolation of T and B cells by CD45 beads provides high yield and purity during muscle regeneration. In addition, myogenic cells can also be isolated from the same samples.

The presence of muscle stem cells is essential to elucidate the mechanism of muscle homeostasis [46,47,48]; therefore, it is necessary to analyze myogenic cells along with immune cells, such as T and B cells. The isolation of T and B cells from skeletal muscle samples using CD45 beads has an advantage. Towards this, we examined whether CD45^−^ cells could also be used to analyze myogenic cells. In muscle samples from wild-type and *mdx* mice obtained on day 3 after injury, CD45^−^ cells, which were not used for T and B cells isolation, were stained with antibodies and analyzed using flow cytometry for muscle cells. The analysis indicated a population of myogenic cells with a sufficient number of isolated cells (Figure 4A,B). In wild-type mice, the percentage of myogenic cells in the CD45-negative cells was 14.1 ± 1.3%, with a yield of 6.4 ± 1.4 × 10^3^ cells (Figure 4A), whereas, in *mdx* mice, the percentage of myogenic cells was 4.6 ± 0.4% and the cell number was 2.1 ± 0.3 × 10^3^ (Figure 4B). Isolated myogenic cells were also confirmed by immunohistology to represent MyoD-positive cells (Figure S1). Taken together, these data clearly demonstrate that in regenerating muscle samples, isolation of T and B cells using CD45 beads is possible with high efficiency in terms of both purity and yield. Furthermore, CD45^−^ cells, obtained from the same samples after CD45^+^ cell separation, led to efficient myogenic cells isolation (Figure 4C).

## 4. Discussion

In this study, we compared the isolation of lymphocytes during skeletal muscle regeneration using Percoll and CD45 beads and evaluated the methods for better isolation, purity, and high yield. We found that the CD45-magnetic bead method was superior in the isolation of T and B cells in regenerating muscle. Furthermore, we found that CD45-negative cells could be used to efficiently isolate myogenic cells from the same samples as the T and B cells, which is an additional advantage of isolation using CD45 beads. In addition to lymphocytes, myogenic cells are essential for muscle regeneration [5,46,49]. Examination of these cells together would provide important insights into the mechanisms of muscle regeneration.

On day 3 of muscle regeneration, T and B cells accumulated to a greater extent in the muscle. This result is consistent with a previous study in which CD4^+^ T cells and CD8^+^ T cells were high on day 3 after injury [22,23]. These results clearly indicate that lymphocytes influence myogenic cell activation during muscle regeneration. However, when muscle tissue is in a normal state or completely healed from injury, these lymphocytic cells disappear [11,50]. Therefore, further studies are needed to clarify the interconnection between myogenic cells and these lymphocytes, as these lymphocytes make a transient contribution during muscle regeneration.

Importantly, compared with the collagenase-treated only and antibody-stained groups, the CD45-bead-isolated and antibody-stained groups were superior in terms of cell collection rate and yield. Isolates obtained from collagenase treatment of muscle contain myofibrillar debris [11]. Flow cytometry analysis showed that the CD45-bead-isolated and antibody-stained group had distinctly less debris than the collagenase-treated-only group (data not shown). The reason for the higher yield may be attributed to the removal of debris by CD45 bead treatment. In muscle regeneration, the CD45-magnetic bead method was more efficient in isolating lymphocytes than the Percoll gradient method. Injured muscles often display a large amount of myofibrillar debris [34]. These myofibrillar debris may have possibly impaired the efficiency of lymphocyte isolation by the Percoll separation method. On the contrary, isolation using CD45 beads was unaffected by myofibrillar debris and allowed the isolation of higher yields of the target lymphocytes. A higher yield of cells would be desirable and effective for the subsequent analysis involving the isolated cells including RNA extraction and cell culture. In a previous study [45], the percentage of myogenic cells to total cells on day 3 after injury was 2.8%. The percentage of them from the CD45-negative fraction in this study was 2.1%, which is a similar level to previous data. Thus, for the analysis of T and B lymphocytes together with myogenic cells during the course of muscle regeneration, the CD45 beads isolation method will be suitable method.

Our results showed that the CD45-magnetic bead method was highly efficient in isolating T and B cells, as well as the myogenic cells during muscle regeneration in wild-type and *mdx* mice models. This method is expected to have a wide range of clinical applications and can aid in further research on muscle regeneration involving lymphocytes and cells.

## Supplementary Materials

The following supporting information can be downloaded at: https://www.mdpi.com/article/10.3390/cells11111754/s1, Figure S1: Immunostaining for myogenic markers in sorted cells.
