# Supplementary material for: Efficient Isolation of Lymphocytes and Myogenic Cells from the Tissue of Muscle Regeneration
Source: Cells. 2022 May 26;11(11):1754. doi: 10.3390/cells11111754 (PMC9179359; doi:10.3390/cells11111754)
Supplement: Supplementary file 1 [file cells-11-01754-s001.zip › cells-1736459-supplementary.pdf]

Supplemental Figure S1

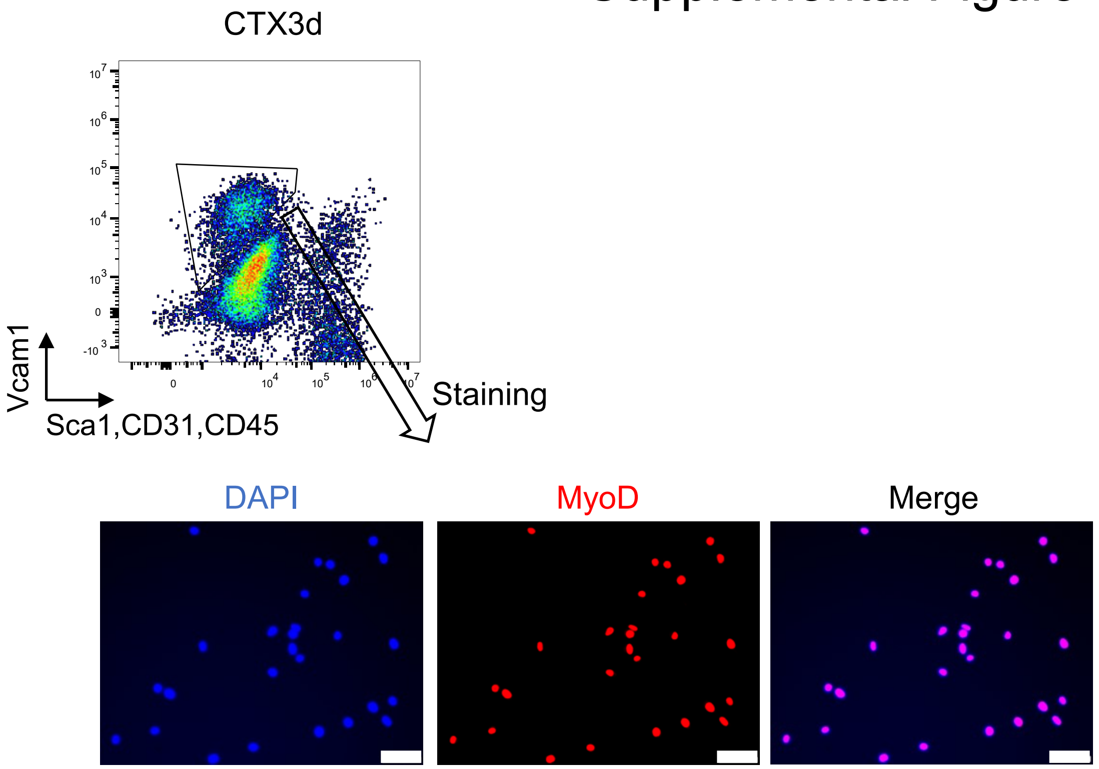

Figure S1. Immunostainig for myogenic markers in sorted cells.
Muscle from wild-type mice on day 3 of muscle injury was enzymatically treated by collagenase treatment. $Vcam1^{+}Sca1^{-}CD31^{-}CD45^{-}$ cells were sorted by FACS and stained with a MyoD (red) antibody. Scale bar, 50 µm.
